# The effect direction plot: visual display of non-standardised effects across multiple outcome domains

**DOI:** 10.1002/jrsm.1060

**Published:** 2012-10-12

**Authors:** Hilary J Thomson, Sian Thomas

**Affiliations:** MRC Social and Public Health Sciences UnitGlasgow, Scotland, G12 8RZ, UK

**Keywords:** data tabulation, multiple outcomes, narrative synthesis, non-statistical heterogeneity

## Abstract

Visual display of reported impacts is a valuable aid to both reviewers and readers of systematic reviews. Forest plots are routinely prepared to report standardised effect sizes, but where standardised effect sizes are not available for all included studies a forest plot may misrepresent the available evidence. Tabulated data summaries to accompany the narrative synthesis can be lengthy and inaccessible. Moreover, the link between the data and the synthesis conclusions may be opaque.

This paper details the preparation of visual summaries of effect direction for multiple outcomes across 29 quantitative studies of the health impacts of housing improvement. A one page summary of reported health outcomes was prepared to accompany a 10 000-word narrative synthesis. The one page summary included details of study design, internal validity, sample size, time of follow-up, as well as changes in intermediate outcomes, for example, housing condition. This approach to visually summarising complex data can aid the reviewer in cross-study analysis and improve accessibility and transparency of the narrative synthesis where standardised effect sizes are not available. Copyright © 2012 John Wiley & Sons, Ltd.

## 1. Introduction

Policy relevant systematic reviews of complex interventions often address broad questions and are not without their challenges (Jackson *et al*., 2004). The data for synthesis are typically highly heterogeneous both statistically and also with respect to included study methods, interventions, contexts, populations and outcomes (Doyle *et al*., 2008) (Shepperd *et al*., 2009). One challenge is managing the complex dataset of multiple outcomes and diverse study characteristics, while preparing a synthesis which is both accessible to potential evidence users and maintains the integrity of the systematic review approach. That is, to present a synthesis that is transparent and reflects the quality of the evidence.

Careful tabulation of reported results is an essential component of synthesis and careful visual display can aid interpretation and access to complex data for both the reviewer and the reader. Tufte (1983) proposes that visual representations should facilitate understanding of large datasets, different levels of detail, comparison of the data, and be closely integrated with accompanying descriptions of the data (Tufte, 1983). The forest plot meets these criteria and is a valuable visual tool regardless of whether the data are to be meta-analysed or synthesised narratively. However, in reviews where standardised effect sizes are not available across the included studies, the use of a forest plot will under-represent the available evidence. Despite advocacy of careful data tabulation to accompany narrative synthesis, there is little guidance and few examples to help reviewers present accessible digests of complex data (Popay *et al*., 2006). Consequently, narrative synthesis can result in a lengthy textual synthesis, and tables that are inaccessible to anyone but the most committed reader.

We recently conducted a systematic review of the health impacts of housing improvements (Thomson *et al*., 2010) (Thomson *et al*., 2011 (submitted)). The review included 29 quantitative intervention studies that had assessed changes in any health or well-being outcome following housing improvement. Study designs included in the review were randomised controlled trials (RCTs), controlled before and after (CBA) studies and uncontrolled studies. The included studies varied in further ways, including intervention components, implementation, context, population, study quality, sample size, outcomes reported and timescale of follow-up. Few studies reported data amenable to calculation of standardised effect sizes limiting the use of a forest plot. This paper details our methods for preparing a visual summary of complex quantitative data to accompany more detailed tables and narrative synthesis.

## 2. Methods

Studies were grouped into four broad intervention categories reflecting the various types of housing improvements included in the review, as well as the timescale and context of the intervention. Outcomes were also grouped into four broad categories: respiratory, mental, general health and other symptoms/illness. Full details of the review inclusion criteria and critical appraisal methods are reported elsewhere (Thomson *et al*., 2010).

Following detailed data extraction, the data were tabulated in different ways representing increasing levels of summary. The studies were ordered by intervention category, overall study quality (incorporating an assessment of potential sources of bias from study design, sample selection, control for confounding and attrition at follow-up), date of publication and study design (RCT, CBA, uncontrolled before and after, cross-sectional CBA, cross-sectional uncontrolled before and after and retrospective uncontrolled study) prioritising experimental and controlled studies and those studies that followed a cohort over time rather than rely on cross-sectional data.

In addition to tables reporting conventional textual descriptions of the data, two further tables were prepared that used visual representations, arrows, to indicate reported effect direction (improvement 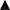, deterioration 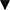, or no change/conflicting findings 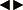). An indication of study size and statistical significance was incorporated in the arrow using size and colour, and superscript was added to indicate the type of statistical test being represented (controlled studies: difference between control and intervention group at follow-up (unless stated); ^a^ difference in change between control and intervention group; ^b^ change within intervention group only. Uncontrolled studies: change since baseline).

One of these tables detailed the individual outcomes reported. A second table using arrows presented a single arrow for each outcome domain per study. Where more than one outcome was reported within an outcome domain, the arrow represented a synthesis of reported impacts directions and statistical significance and subscript was used to indicate the number of outcomes being synthesised in the single arrow. The criteria for developing the “synthesised” arrows of effect direction were developed as follows:

- Where multiple outcomes all report effects in the same direction and with the same level of statistical significance:
  - report effect direction and indicate overall level of statistical significance.
- Where direction of effect varies across multiple outcomes:
  - report effect directions and statistical significance where 70% of outcomes report similar direction and similar statistical significance.
  - if <70% of outcomes report consistent direction of effect report no clear effect/conflicting findings 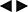 (size to reflect sample size).
- Where statistical significance varies across more multiple outcomes:
  - if effect directions similar AND >60% outcomes statistically significant, report as statistically significant (black arrow).
  - if effect directions similar AND <60% outcomes statistically significant, report as not statistically significant (grey arrow).

Where space allowed the tables incorporated a description of study design, change in the intermediate outcome (i.e. housing condition), size of intervention and control group at follow-up, time point of assessed outcome and intervention implementation.

The tables with the arrows were prepared by one reviewer (HT) and checked by a second reviewer (ST). The tables were prepared in Microsoft Word.

## 3. Results

Web Tables 1 and 2 show examples of the conventional tabulation of textual data. These tables include different levels of detail regarding internal validity items and other study details. Table 1 presents the reported effect directions for each individual outcome as arrows for the same example study, with all 29 studies and included summaries of relevant sub-group analyses. This table is six pages (A4) long (see web Table 3 for the full version).

**Table 1:** Visual representation of reported effect direction for individual outcomes (*NB: Arrow size denotes study size not effect size*)

Intervention: warmth and energy efficiency improvements (post 1980)									

Author year	Study grade	Housing condition	General health		Respiratory		Mental	Illness/symptoms	
Experimental studies									
Howden-Chapman *et al*., 2008 (children)	A	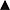	Poor/fair health	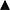	Sleep disturbed by wheeze	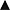		Diarrhoea	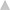
					Wheeze limits speech	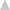		Twisted ankle	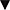
					Wheeze during exercise	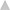		Vomiting	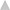
					Dry cough at night	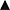		Ear infection	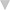
					Cough at night (diary)	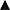			
					Cough on waking (diary)	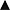			
					Cough during day (diary)	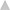			
					Cough overall	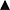			
					Lower resp symptoms	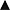			
					Upper resp symptoms	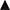			
					Wheeze overall	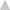			

Sample size: final sample size (individuals) in intervention group large arrow >300; medium arrow 50–300; small arrow <50.

Effect direction: upward arrow = positive health impact, downward arrow = negative health impact, sideways arrow = no change/conflicting findings.

Statistical significance: black arrow *p* < 0.05; grey arrow *p* > 0.05; empty arrow = no statistics/data reported.

Statistical tests: controlled studies (including RCTs)—difference between control and intervention group at follow-up (unless stated); ^a^ difference in change between control and intervention group; ^b^ change within intervention group only; uncontrolled studies: Change since baseline (unless stated).

Table 2 presents a one page summary (in the original Word document this table fitted onto one page) of 29 studies with respect to reported effect direction for the broad outcome categories. The studies are grouped according to the broad intervention category and ordered by overall study quality (column 3), date of publication and study design, placing those studies judged to represent the best available and most recent evidence at the top of each intervention category.

**Table 2:** Summary of direction of health impacts from included studies (*NB: Arrow size denotes study size not effect size*)

Author year	Study design	Study quality	Final sample int/cont	Time since interv'n	Housing condition	Interv'n integrity	General health	Respiratory	Mental	Illness/symptoms
Intervention: warmth and energy efficiency improvements (post 1980)										
Osman *et al*., 2010	RCT	A	45/51	5 months	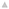	C	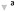	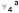		
Howden-Chapman *et al*., 2008 **	RCT	A	175/174	4–5 months	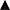	C	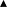	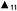		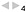
Braubach *et al*., 2008	CBA	A	∼210/165	5–8 months	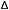	C	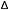	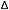	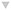	
Barton *et al*., 2007 *, ****	RCT	A	193/254	3–10 months	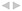	C	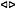	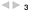	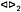	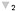
Howden-Chapman *et al*., 2007 *	RCT	A	1689/1623	<1 year	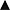	C	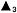	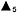	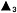	
Platt *et al*., 2007	CBA	A	1281/1084	1–2 years	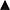	B	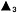	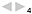	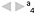	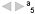
Lloyd *et al*., 2008	CBA	B	9/27	1–2.5 years		C				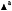
Shortt *et al*., 2007	CBA	B	46/54	1–3.5 years	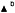	C		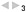	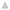	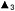
Somerville *et al*., 2000 **	UBA	B	72	3 months	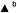	B		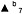		
Hopton *et al*., 1996 **	CBA	B	55/77	5–11 months	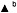	C		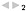	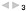	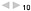
Allen 2005	UBA	C	16	<1 year	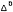	C			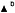	
Allen 2005 a	UBA	C	24	<3 years	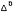	C	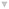		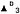	
Health Action Kirklees 2005	R	C	102	2–8 months	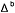	B				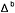
Iversen *et al*., 1986	CBA	C	106/535	3–6 months	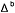	B		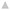		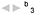
Intervention: rehousing/retrofitting +/− neighbourhood renewal (post 1995)										
Kearns *et al*., 2008 **	CBA	A	262/284	24 months	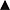	C	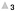	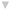	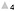	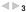
Thomson *et al*., 2007	CBA	A	50/50	12 months	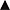	B	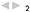		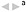	
Critchley *et al*., 2004	CBA	A	∼109/137	1–12 months	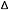	B	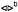		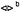	
Thomas *et al*., 2005	CBA	B	585/759	22 months	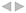	C			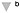	
Barnes *et al*., 2003	CBA	B	45/45	18 months	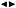	C	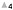		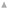	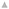
Evans *et al*., 2002	CBA	B	17/17	6–18 months	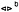	C	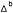			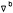
Blackman *et al*., 2001 *	UBA	C	166	5 years	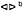	C	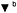	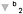	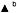	
Wells 2000	UBA	C	23	2–3 years	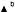	B			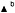	
Ambrose 1999	UBA	C	227	4 years	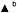	C		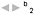	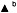	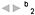
Halpern 1995	XUBA	C	27	10 months		C			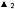	
Intervention: provision of basic housing needs/low or middle income country intervention										
Spiegel *et al*., 2003	XCBA	C	896/807	1–4 years	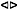	C	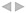			
Aziz *et al*., 1990 **, ****	XCBA	C	∼ > 200/200	2–3 years	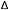	B				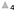
Intervention: rehousing from slums (pre 1965)										
Wilner *et al*., 1960	CBA	A	1891/2893	<1 year	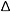	B			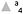	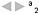
Chapin 1938	UBA	C	171	8–19 months	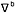	B			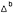	
McGonigle *et al*., 1936 *, ***	XCBA	C	<152/289	5 years	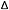	C				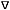

* data for children also available

** children only

*** area level data not relating to study population alone

**** adults and children aggregated

Study design: RCT, randomised controlled trial; CBA, controlled before and after study; UBA, uncontrolled before and after study; XCBA, cross-sectional controlled before and after study; XUBA, uncontrolled cross-sectional before and after study; RC, retrospective controlled study (recall of change in health outcomes after intervention); R, uncontrolled retrospective study.

Effect direction: 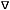
= positive health impact, 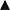
= negative health impact, 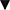
= no change/conflicting findings

Sample size: final sample size (individuals) in intervention group large arrow >300; medium arrow 50–300; small arrow <50.

Statistical significance: black arrow *p* < 0.05; grey arrow *p* > 0.05; empty arrow = no statistics/data reported.

Statistical tests: controlled studies—difference between control and intervention group at follow-up (unless stated); ^a^ Difference in change between control and intervention group; ^b^ Change within intervention group only.

Uncontrolled studies: change since baseline (unless stated).

Number of outcomes within each category synthesis is 1 unless indicated in subscript beside effect direction.

Synthesis of multiple outcomes within same outcome category

Where multiple outcomes all report effect in same direction and with same level of statistical significance, report effect direction and indicate overall level of statistical significance

Where direction of effect varies across multiple outcomes:

Report direction of effect and statistical significance where 70% of outcomes report similar direction and similar statistical significance.

If <70% of outcomes report consistent direction of effect report no clear effect/conflicting findings (size to reflect sample size)

Where statistical significance varies:

If direction of effect similar AND >60% outcomes statistically significant, report as statistically significant (black arrow).

If direction of effect similar AND <60% outcomes statistically significant, report as not statistically significant (grey arrow).

## 4. Discussion

We have developed visual displays of complex data to accompany a textual narrative synthesis that was over 10 000 words in length. The conventional tables reporting data (web tables 1 and 2) were over 50 and 12 pages long, respectively (Thomson *et al*., 2010).

The data summarised in the visual display or graphical tables described in this paper are highly heterogeneous, yet the tables incorporate a considerable number of key study characteristics, and all tables include an indication of overall study quality. In addition to the display of the wide range of primary health outcomes, the tables incorporate an indication of intermediate outcomes, changes in housing outcomes, which may shed light on why effects were or were not observed. The intermediate outcomes can be selected to reflect pre-specified steps in the logic model or theory used to shape the review (Anderson *et al*., 2011). The longer table (Table 1) includes sub-group analysis reflecting data reported in the text of the synthesis. Sub-group analysis may also be included in the “within-study” synthesis table (Table 2). The table allows for flexibility in what is included and the order of the studies. Re-ordering of the studies may be useful when performing preliminary synthesis, and the content and order of the table may vary depending on the purpose of the table. In addition to presenting a clear visual summary of the effect directions, the table also facilitates identification of where there is a lack or absence of evidence; the gaps in the table allow immediate identification of outcome domains with few or no data.

Because of the lack of standardised effect sizes across the outcomes, it is not possible to present an indication of effect size. The arrow size that represents study size has potential to be misinterpreted as effect size, hence the need to add a note in the title of the table. The effect direction plot is not intended to replace reporting of standardised effect sizes. Rather, the effect direction plot is an additional tool to allow representation of all included data whether or not standardised effects are available. The single arrow used to represent multiple similar outcomes presents a single visual to represent multiple outcomes within an outcome domain. Although our approach aims to be transparent and systematic, the criteria used to represent study size and synthesise multiple similar outcomes from single studies is arbitrary, and the visual representation should only be interpreted as indicative. The possibility of using the effect direction plot to “vote count” as a form of synthesis should clearly be warned against. However, we have no reason to think that the effect direction plot is more likely to be used to “vote count” than a forest plot of the standardised effect sizes and confidence intervals of individual studies. Indeed, the presentation of the multiple outcomes and study characteristics may draw attention to the complexity of the data and thus the hazards of vote counting.

The value of careful data visualisation in promoting analysis and access to complex data is well established and used across all disciplines. Some early examples of their development and use including Florence Nightingale's rose diagram that was used to highlight the impact of insanitary conditions in the military, (Cohen, 1984) and John Snow's mapping of cholera cases to investigate the route of transmission (Rosen, 1993). Within the systematic review field, statistical graphics such as the forest plot and the funnel plot are very valuable but are limited to representing standardised effect sizes. Few tools are available to help reviewers present accessible digests of complex data, for example, where multiple outcomes are included reviews, or where standardised effects sizes are not available. Ogilvie and colleagues have previously developed the harvest plot to present complex data on differential effects and associations (Ogilvie *et al*., 2008; Wijndaele *et al*., 2009). This is a valuable development particularly where reviews investigate impacts on inequalities between groups. The effect direction plot provides analytic support to the reviewer by facilitating cross-study comparison of effect directions. The plots are of particular use in reviews that include multiple outcomes and where forest plots may under-represent the data. The effect direction plot provides clear data summaries for the reader as well improving the transparency of the link between the lengthy textual narrative analysis of the studies and the conclusions of the narrative synthesis (Lucas *et al*., 2007). We acknowledge the limitations of the effect direction plots and would be keen to hear readers' thoughts on how to improve them, for example, establishing agreed criteria and development of machine readable codes to produce the graphic.

## Supplementary material

The supporting information may ^b^e found in the online version of this article
