# Supplementary material for: The effect direction plot: visual display of non-standardised effects across multiple outcome domains
Source: Res Synth Methods. 2012 Oct 12;4(1):95–101. doi: 10.1002/jrsm.1060 (PMC3688329; doi:10.1002/jrsm.1060)
Supplement: Supplementary file 1 [file jrsm0004-0095-SD1.doc]

**Table web 1: Summary of data extraction- methods & results**

**Author/Year/Reference:** Howden-Chapman et al 2008

**Location:** New Zealand

**Overall Study Grade:** A

**Study population/context**: Four New Zealand cities. Households with child (6-12 years) with Dr diagnosed asthma in house with main form of heating plug in heater or unflued LPG heater. Mean age 9.6 years, ~58.5% male, ~36.5% Maori (compared to 15% general population), 47% NZ European Int/Cont.

**Intervention category:** Warmth and energy efficiency improvements (after 1980)

**Intervention description:** Replacing 2kW electric heaters or portable unflued gas heaters with ≥4kW non-polluting alternative.

Choice of 3 heaters: 131 (73.6%) heat pump, 39 (21.9%) wood pellet burner or 5 (2.8%) flued gas heater. (No indication of proportion of each intervention by Int & Cont group). All homes were (where necessary) brought up to the NZ building code standard before baseline data collection.

*Was intervention group distinct from control group in terms of housing changes?* Yes

*Variation in intervention types delivered to intervention group:* some

*Variation in extent of housing improvement reported by participants:* Not reported

**Summary of intervention integrity: C**

*Study design (in relation to reported health outcomes)*: Randomised controlled trial

*Method of randomisation****:*** NA

**Summary of study design: A**

*Selection of sample:* Identified eligible families through primary care staff and radio adverts

*Baseline response rate*: Not reported

**Summary of selection bias: C**

*Final sample size:* Final/Baseline: 349/409 (85.3%) children. Int 175/200 (87.5%) Cont 174/209 (83.3%)

*Difference between responders and non-responders:* Not reported

**Summary of withdrawals: A**

*Data collection methods:* Interviews with parents plus diaries of child asthma symptoms, FEV & PEF on electronic PIKO meters

*Methods/tools piloted:*

*Health outcomes reported:* Peak flow, FEV, LRS, URS, cough (various measures), use of inhalers, wheeze, diarrhoea, vomiting, infections, twisted ankle, Health service use related to asthma, days of school.

**Summary of data collection: A**

*Similarities between control and intervention group demonstrated*: Gender, age, ethnicity, parental history of asthma, exposure to tobacco smoke in house, presence of unflued gas heater in house, housing conditions (all houses brought to New Zealand insulation standard before study)

*Key confounders were adjusted for in analysis:* Baseline value for outcome being analysed

**Summary of confounding: A**

*Participants or assessor blinded to intervention allocation:* No

**Summary of blinding: C**

**Follow-up time(s)** (TO = baseline T1 = first follow up)**:** Once: 4-5 months since intervention. 12 months since baseline (both data collection times over 4 winter months, June-September).

| Summary of Cochrane Risk of Bias Items | | | | | | | | | | | | |
| --- | --- | --- | --- | --- | --- | --- | --- | --- | --- | --- | --- | --- |
| Random sequence generation (selection bias) | Allocation concealment (selection bias) | Blinding of participants and personnel (performance bias) | Blinding of outcome assessment (detection bias) | Blinding of analysts | Incomplete outcome data (attrition bias) | Selective reporting (reporting bias) | Baseline outcome characteristics similar | Baseline characteristics similar | Contamination | Baseline response | Attrition | Implementation of intervention |
| ? | L | ? | ? | ? | L | ? | L | L | ? | ? | L | ? |

**Results**

Health: (Int/Cont n=175/174)) (OR for Int group adjusted for baseline measure where available) (95% CI) Parent reported measures- poor/fair health (as opposed to good/very good/excellent) (n=346) OR 0.48 (0.31 to 0.74), p<0.001; sleep disturbed by wheeze (n=344) OR 0.55 (0.35 to 0.85), p<0.001; wheeze limits speech (n=344) OR 0.69, (0.40 to 1.18) p=0.18; wheeze during exercise (n=344) OR 0.67 (0.42 to 1.06) p=0.09; dry cough at night (n=345) OR 0.52 (0.32 to 0.83), p=0.01; (included as dummies- diarrhoea (n=343) OR 0.72 (0.45 to 1.16), p=0.18; vomiting (n=344) OR 0.88 (0.55 to 1.40), p=0.58; ear infections (n=344) OR 1.16 (0.68 to 1.99), p=0.58). Asthma diary (Int/Cont n=~175/174) (adjusted for baseline value) Mean Ratio (MR: mean score Int divided by Cont) (95% CI) cough at night (n=352) MR 0.72 (0.59 to 0.89), p=0.002; cough on waking MR 0.67 (0.53 to 0.84), p<0.001; cough during the day MR 0.84 (0.70 to 1.01), p=0.06; cough overall (n=349) MR 0.75 (0.62 to 0.92), p=0.005; wheeze overall (n=345) MR 0.67 (0.50 to 0.91), p=0.01; lower respiratory tract symptoms (n=345), MR 0.77 (0.73 to 0.81), p=0.01; upper respiratory tract symptoms (n=360) MR 0.92 (0.74 to 1.14), p=0.43. Lung function measures (Int/Cont n=~175/174) (effect size- beta adjusted for baseline value (95% CI)) PEFR morning (n=347) b=+8.92, (-7.66 to +25.50), p=0.29; FEV1 morning (n=346) b=+57.0 (-75.4 to +189.4), p=0.4; number of preventer inhaler (beclamethasone) (n=363) MR 1.08 (0.67 to 1.74), p=0.74; use of reliever inhaler (salbutamol) (n=364) MR 0.55 (0.44 to 1.05), p=0.08. Mean for Int compared with Cont (adjusted for baseline value) (95% CI): (n=333) asthma visits to GP (n=323) -0.40 (-0.62 to 0.11), p=0.01; other visits to GP (n=333) -0.27 (-0.46 to -0.01), p=0.04; asthma visits to nurse (n=335) -0.05 (-0.2 to 0.24), p=0.67. Twisted ankle 1.86 (1.03 to 3.35) (unadjusted).

Housing: At TI Mean temperature over 4 winter months (oC)- living room Int v Cont 17.07 v 15.97, p<0.001 (95% CI 0.54 to 1.67); child’s bedroom 14.84 v 14.26, p=0.03 (95% CI 0.05 to 1.08); degree hours per day <10oC (hours per day multiplied by number of degrees below 10oC) 1.13 v 2.31, p=0.001 (95% CI 0.49 to 1.93); hours per day <10oC in child’s bedroom 2.03 v 4.29, p<0.001 (95% CI 0.99 to 2.34). Mean NO2 over one month- in child’s bedroom (μg/m3) (Int v Cont) 7.3 v 10.9, p<0.001; living room NO2 8.5 v 15.7, p<0.001 (outdoor NO2 levels unchanged).

Other: Mean for Int compared with Cont (adjusted for baseline value) (95% CI): days off school (parent reported) (n=333) -0.73 (-1.94 to 0.67). Mean school absence (days of absence reported by school) Int/Cont 7.6/9.6, effect ratio 0.79 (95% CI 0.66 to 0.96). Sub-group analysis reported greater effect ratio for those whose pre-intervention heat source was an unflued gas heater (compared to an electric heat source) effect ratio 0.72 (95% CI 0.55 to 0.93)

**Table web 2: Tabulated summary of study quality and results**

**Intervention type: Warmth/energy efficiency measures**

| Author, publication year, country, reference | Study design, final sample size, number and times of follow-up | Summary | | | | | | | | Summary of results |
| --- | --- | --- | --- | --- | --- | --- | --- | --- | --- | --- |
|  |  | Selection | Confounding | Withdrawals | Data coll’n | Blinding | | Int integrity | Overall grade |  |
| Heyman et al,  *(subm)*  UK | Randomised controlled trial  Final/Baseline sample:140/237 (59%)  Twice: 6-12 & 18-24 months since intervention | B | B | C | A | | C | C | A | Health: Authors report no evidence of health impact with respect to SF-36, self-reported health, and self-reported health service use after the intervention- no data reported.  Housing Ŧ Φ: Mean SAP Before (Yr 2) (Int/Cont n=114/92) 46.7/48.9 ; Time I 61.1/48.5 (n=96/82). Mean evening living room temperature (oC) in winter (Time I Int/Cont n=48/48)14.5/13.1*; mean fuel expenditure (Time I Int/Cont n=99/83) £596/£567, p=0.408. Change mean warmth satisfaction score (4 point scale) Before (Yr 2)-After (Yr 3) Int/Cont (n~96/82) +1.18/+0.64**. |
| Howden-Chapman et al,  2008  New Zealand | Randomised controlled trial  Final/Baseline: 349/409 (85.3%) children  Once: 4-5 months since intervention. 12 months since baseline | C | A | A | A | | C | C | A | Health Φ: (OR for Int group adjusted for baseline measure where available) (95% CI) Parent reported measures- poor/fair/good health (as opposed to very good/excellent) (n=346, ~50% Int group) OR 0.48 (0.31 to 0.74)***; sleep disturbed by wheeze (n=344) OR 0.55 (0.35 to 0.85)**; wheeze limits speech (n=344) OR 0.69, (0.40 to 1.18); wheeze during exercise (n=344) OR 0.67 (0.42 to 1.06); dry cough at night (n=345) OR 0.52 (0.32 to 0.83)*; diarrhoea (n=343) OR 0.72 (0.45 to 1.16). Asthma symptom data from diary (Int/Cont n=178/182) (adjusted for baseline value) Mean Ratio (MR: mean score Int divided by Cont) (95% CI) cough at night (n=333) MR 0.72 (0.59 to 0.89)**; cough on waking MR 0.67 (0.53 to 0.84) ***; cough during the day MR 0.84 (0.70 to 1.01). Mean for Int compared with Cont (adjusted for baseline value) (95% CI): days off school (n=333) -0.73 (-1.94 to +0.67), p=0.28; asthma visits to GP (n=323) -0.40 (-0.62 to +0.11) *; other visits to GP (n=333) -0.27 (-0.46 to -0.01)*.  Housing: At TI Mean temperature over 4 winter months (oC)- living room Int v Cont 17.07 v 15.97, p<0.001 (95% CI 0.54 to 1.67); child’s bedroom 14.84 v 14.26, p=0.03 (95% CI 0.05 to 1.08); degree hours per day <10oC (hours per day multiplied by number of degrees below 10oC) 1.13 v 2.31, p=0.001 (95% CI 0.49 to 1.93); hours per day <10oC in child’s bedroom 2.03 v 4.29, p<0.001 (95% CI 0.99 to 2.34). Mean NO_2_ over one month- in child’s bedroom (μg/m3) (Int v Cont) 7.3 v 10.9, p<0.001; living room NO_2_ 8.5 v 15.7, p<0.001 (outdoor NO_2_ levels unchanged). |
| Barton et al,  2007  UK | Randomised controlled (stepped wedge)  Final/Baseline sample: 426/481 (88.6%)  Twice: Total Follow-up maximum of 2 years since intervention | A | A | A | A | | C | C | A | Health Φ: (Time I Int/Cont n=193/254) Int/Cont (TI) change in prevalence of asthma -7%/-3%, ns, OR (95% CI) ~0.95 (0.60 to 1.50); bronchitis +4%/0%, ns, OR ~1.00 (0.48 to 2.13); 'other respiratory' (includes bronchitis but not asthma) -1%/+4%, ns, OR ~1.00 (0.55 to 1.80); arthritis 0%/-2%, ns, OR ~1.31 (0.73 to 2.34); rheumatism +3%/+2%, ns, OR ~0.52 (0.16 to 1.67). Paired analysis (Int/Cont n=14/13 adults, n=25/27 children) No significant difference in changes (Before-After(TI) Int/Cont) for six individual respiratory symptoms; summed score of six respiratory measures: adults -2.3 v +1.1, p=0.006, children -1.8/-1.0, p=0.17.  Housing Φ: Change (Before-AfterTI) mean temperature (oC) (bedroom) (Int/Cont n=49/69) Int v Cont +2 v +1, (living room) 0 v 0. No significant changes in environmental measures of air quality- particles (coarse and fine) or airborne microbes or relative indoor humidity. |
